# Locally-tailored vs. centrally-administered strategies for implementation of primary human papillomavirus (HPV) screening in an integrated healthcare system: a qualitative research study

**DOI:** 10.3389/frhs.2025.1595934

**Published:** 2025-07-15

**Authors:** Erin E. Hahn, Corrine Munoz-Plaza, Chunyi Hsu, Nancy T. Cannizzaro, Quyen Ngo-Metzger, Michael K. Gould, Brian S. Mittman, Melissa Hodeib, Devansu Tewari, Chun R. Chao

**Affiliations:** ^1^ Department of Research and Evaluation, Kaiser Permanente Southern California, Pasadena, CA, United States; ^2^ Department of Health Systems Science, Kaiser Permanente Bernard J. Tyson School of Medicine, Pasadena, CA, United States; ^3^ Department of Obstetrics and Gynecology, Southern California Permanente Medical Group, Riverside, CA, United States; ^4^ Department of Obstetrics and Gynecology, Southern California Permanente Medical Group, Irvine, CA, United States

**Keywords:** implementation strategies, cervical cancer screening, primary HPV screening, qualitative research, healthcare delivery system change

## Abstract

**Introduction:**

Primary human papillomavirus *(*HPV*)* testing is recommended for cervical cancer screening for women aged 30–65 years without a history of abnormal results. However, there is little clear guidance regarding effective strategies for implementing primary HPV screening. As part of an ongoing randomized trial comparing implementation strategies for primary HPV testing (a centrally administered + usual care strategy vs. centrally administered + locally tailored strategy), we evaluated clinician experiences and perceptions of large-scale implementation of primary HPV screening in an integrated healthcare system, Kaiser Permanente Southern California.

**Materials and methods:**

We conducted qualitative interviews with internal medicine, family medicine and obstetrics/gynecology clinicians to gain insight into fidelity to the interventions and implementation strategies, barriers and facilitators to implementation, and recommendations. Participants from both arms of the trial were recruited. Interview guides were developed with the Consolidated Framework for Implementation Research (CFIR). We recruited physicians, licensed vocational nurses, and medical assistants after primary HPV screening had been implemented. Interviews were recorded and transcribed. Using a team coding approach, we developed an initial coding structure refined during iterative analysis; data were subsequently organized thematically into domains, key themes, and sub-themes using thematic analysis, followed by framework analysis informed by CFIR.

**Results:**

Thirty-two interviews were conducted. Participants in both arms of the trial noted high awareness, preparedness, buy-in, and fidelity to the new screening process. Initial barriers concerned specimen collection, proper ordering, and lab delays. An unanticipated barrier was the length of time needed to return lab results for reflexive cytology tests after a positive HPV result which reportedly increased patient anxiety. Participants in both arms reported fidelity to the centralized strategy (e.g., attending webinars, leadership announcements). In the local-tailored arm, few participants recalled the local-tailored resources.

**Discussion:**

The centralized strategy was perceived as highly acceptable and feasible, and fidelity to the associated interventions appear to be facilitators of practice change. Recommendations for improving implementation included patient education, outreach and ongoing clinician training. Findings can be applied to other health systems and settings considering primary HPV screening implementation, particularly those within the U.S. or with a similar health care model.

**Trial Registration:**

ClinicalTrials.gov, identifier #NCT04371887

## Introduction

Within the United States, primary human papillomavirus (HPV) testing is now recommended for cervical cancer screening for women aged 30–65 years with recommendations from the United States Preventive Services Task Force (USPSTF) (1) and other professional societies (2–4). These recommendations are based on studies demonstrating that primary HPV testing is superior to cytology alone (Papanicolaou testing, or Pap) (5) and is as effective as co-testing (Pap plus HPV test) (6–8). Many healthcare organizations are preparing to switch from the previously recommended strategies of co-testing or Pap alone and initiate primary HPV testing, as has been done in several other countries including the Netherlands, Turkey, Canada, England, and Australia (9–12).

Previous research has investigated providers' perceptions and knowledge of the HPV test and primary HPV screening. Studies have found that a variety of clinicians in varying roles viewed primary HPV testing positively (13, 14), knowledge-level among clinicians predicts willingness to accept primary HPV testing (15), and strategies are needed to increase provider knowledge regarding primary HPV screening test effectiveness and guideline-recommended screening intervals (16). Patients' perceptions of primary HPV testing are often less accepting (17–19), with research suggesting that increasing women's knowledge about HPV and HPV testing may facilitate the acceptability of primary HPV testing (20), including the development of patient-directed communication which highlights the effectiveness of primary HPV screening (19).

Based on these experiences and modeling studies, the transition to primary HPV testing is expected to result in improved cost effectiveness and simplified clinical care processes for those converting from co-testing to primary HPV testing (e.g., one sample taken instead of two at point of care) (21, 22). However, there is little clear guidance regarding effective strategies for achieving optimal implementation and stakeholder-centered outcomes. Within the United States, the decision to transition to primary HPV screening is mainly left to individual health systems rather than implemented on a national or state level. In 2020, Kaiser Permanente Southern California (KPSC), a large integrated health system, enacted a system-level transition from HPV co-testing to primary HPV testing for women aged 30–65 in response to the updated USPSTF guidelines.

The overarching goal of the main trial was to compare two implementation strategies for primary HPV testing: a “centrally-administered + usual care” strategy (hereafter referred to as “centrally-administered”) vs. “centrally-administered + locally-tailored” strategy (hereafter referred to as “locally-tailored”). The centrally-administered strategy that all sites received included clinician-level interventions such as education (e.g., training webinars, announcements from local and regional leadership) as well as system-level interventions including infrastructure changes (e.g., laboratory preparedness, electronic medical record alerts and cervical cancer screening order redesign). The locally-tailored strategy added adaptable interventions such as additional clinician education and patient educational materials for clinician-patient communication tailored to local clinic needs and context. We were guided by the core function/form framework as the basis for complex health intervention adaptation and tailoring (23–26). Clinic teams at clinics in the locally-tailored arm of the trial were convened to rank needs/barriers followed by review and discussion of rankings and selection of locally-tailored intervention options. A detailed description of the clinical trial protocol has been previously published (27) as well as the main findings (28).

This manuscript presents the post-implementation qualitative research design, recruitment efforts, materials and methods, and qualitative thematic findings.

## Materials and methods

### Study design

As part of a cluster randomized trial evaluating implementation strategies to facilitate primary HPV testing, we conducted a series of post-implementation semi-structured qualitative interviews with KPSC clinicians to evaluate perceptions and experiences with the implementation strategies and interventions. Our findings are reported using the criteria for reporting qualitative research (COREQ) (29). The checklist can be found in Additional File 1.

### Study setting and participants

KPSC is an integrated healthcare delivery system serving over 4.4 million members. Members are racially, ethnically, and socioeconomically diverse and broadly representative of the underlying Southern California population (30). The transition to primary HPV testing was implemented in mid/late 2020, with educational interventions concluding in late fall 2020. We conducted interviews with participants from the centrally-administered arm of the trial starting early 2021. Clinicians from the tailored arm were recruited in early 2022, after the tailoring activities and tailored-intervention delivery were complete (summer-fall 2021). We invited clinicians practicing in settings affected by the practice change in internal medicine, family medicine, and obstetrics/gynecology clinics, including physicians, licensed vocational nurses (LVNs), and medical assistants (MAs). We obtained verbal consent from all participants, which was approved by the KPSC Institutional Review Board [IRB #12015].

### Interview guide and conceptual model

We conducted interviews both prior to the practice change (pre-implementation) to gain insight into perceived barriers and recommendations and after the implementation period had concluded (post-implementation). Details on the baseline interviews have been previously published (14). Here we present our post-implementation qualitative findings from clinician interviews, which included stakeholders from both arms of the trial. The interviews focused on reflection and evaluation of their care transition experience, following guidance from the Consolidated Framework for Implementation Research (CFIR), specifically the “Implementation Processes' domain (46).

Interviews were facilitated using semi-structured interview guides. The overall goal of the interviews was to obtain reflections, evaluation, and experiences with the primary HPV screening implementation over time, after the initial implementation period had concluded. We also assessed awareness of and fidelity to the implementation strategies. For this study, we refer to the term “fidelity” to describe reported stakeholder receipt, awareness, and use of the implementation strategies (e.g., messaging and materials provided by either the centrally-administered or locally-administered strategies in preparation of the transition to primary HPV screening), as well as fidelity to conducting primary HPV screening (e.g., did they transition away from co-testing to adoption of primary HPV testing).

The guides included open-ended questions based on central research questions and probing questions, which allowed for deep exploration of participant responses and probes for emergent themes. Our iterative interview guide development was informed by the CFIR (31). The CFIR is widely recognized to provide a comprehensive overview of relevant constructs applicable to implementation research and clinical practice change (32). We developed potential interview questions based on the HPV testing and implementation science literature and mapped the question domains to relevant CFIR constructs. The interview guide was further discussed and refined by the study team after the initial 2–3 interviews.

Domains in the interview guides included the following: (1) pre-implementation resources and training (e.g., “Thinking back, what primary HPV testing educational and/or training resources were you provided before the change to HPV primary testing?”, “To what extent do you believe you were sufficiently trained and prepared to personally help implement this practice change?” (2) intervention implementation—fidelity and team feedback/engagement (e.g., “Has your team fully adopted the change from co-testing to HPV primary testing?” (3) barriers and facilitators to implementation (e.g., “Overall, how easy or difficult was changing from co-testing to HPV primary testing?”, “How have patients reacted to this practice change?” (4) adaptations to recommended care process required to change from co-testing to HPV primary testing (e.g., “Did your team make any changes or adaptations to the recommended care processes required to change from co-testing to HPV primary testing?”). The domains and questions in the interview guides for both the tailored and usual care arm were the same. The interview guide is available in Additional File 2.

### Recruitment

To attain theoretical data saturation, we planned 25–35 total interviews. We emailed invitations (including a study information sheet describing the research team's interest in the research topic and interview format) and three reminders to approximately 1,300 clinicians assuming anywhere from a 2%–10% positive response and, of those, 20%–50% who would schedule, and complete interviews based on our prior experience in conducting qualitative research with these 7–8 stakeholder groups (33–35). No prior relationships existed between the interviewers (CMP, NTC, CH) and participants prior to study commencement. Participants were informed about the interviewers' role as qualitative researchers on the study team, who had an interest in improving cervical cancer screening practices.

### Interview procedures

We conducted one-time individual semi-structured interviews. Interviews were conducted over the telephone or via Microsoft TEAMS by CMP, NTC, and CH. Interviews lasted approximately 30–60 min and were digitally recorded and transcribed verbatim into written transcripts as preparation for coding and analysis. We halted further interviews once we were confident that we reached thematic saturation with our interview sample whereby we were no longer eliciting new pertinent information or themes from additional interviews (36–39). Transcription was conducted by an institutional approved vendor. We collected gender and profession data from KPSC's clinician database.

### Coding and analysis

Three research team members with experience in qualitative research (CMP, CH, EEH) coded, organized and analyzed the resulting qualitative data. Analysis followed the table-based matrix approaches as described by Miles and Huberman (40), which allows for pattern analysis of responses. This facilitated identification of items related to fidelity, barriers and facilitators to implementation, use of adaptations, and specific recommendations for refinement. All analysis was conducted using NVivo qualitative software (© QSR International 2020).

We identified a lead coder (CMP) with over 25 years' experience in qualitative data collection and analyses and two secondary coders (EEH and CH) to conduct team coding and analysis. We developed an initial coding hierarchy, which was reviewed and modified iteratively throughout the analysis. All coding development steps were tracked and reported in a codebook, which contributed to the rigor and transparency of the process (41). Coding team members independently coded a random sample of 3 transcripts from both the centralized and the locally-tailored sites. Coders then met several times to compare the independently coded transcripts and analytical memos, work through divergent views of the textual analysis, and achieve consensus. From there, the lead coder coded the remaining transcripts and once that step was complete, the coding team reconvened to finalize the analysis, producing a number of summary documents categorized into domains and primary and secondary themes (i.e., classification trees, quotes-by-theme tables, etc.). Our team did capture brief notes to share with the study team in real-time, these notes were not included as part of the formal analysis, and we did not share transcripts or summary data with the interviewees.

## Results

### Participant characteristics

Thirty-two clinicians completed interviews. Table 1 provides a breakdown of the roles and characteristics. Most participants (88%) were female, 50% were physicians, 44% LVNs, and 6% MAs. Of the thirty-two individuals interviewed, 56% were from the centralized sites and 44% from the locally-tailored sites (Table 1).

**Table 1:** Clinician participant characteristics (*n* = 32).

Participant demographics	(*n*)	(%)
Gender		
Female	28	88
Male	4	12
Role		
Internal Medicine Physician	2	6
Family Medicine Physician	9	28
OB-GYN Physician	5	16
Internal Medicine Licensed Vocational Nurse	2	6
Family Med Licensed Vocational Nurse	8	25
Family Medicine Medical Assistant	1	3
OB-GYN Licensed Vocational Nurse	4	13
OB-GYN Medical Assistant	1	3
Study Condition		
Centralized (control)	18	56
Locally tailored	14	44

### Thematic findings

Participants reflected on the feasibility and acceptability of the practice change, fidelity to the intervention and implementation strategies, barriers to implementation, and use of adaptations (see detailed quotes included in Table 2). Participant recommendations for improving implementation are summarized in Table 3.

**Table 2:** Providers' post-implementation perspectives about the transition to primary HPV cervical cancer screening for patients with a normal screening history, aged 30–65 years.

Themes: Feasibility & Acceptability of the Practice Change
Representative Quotes
Acceptability
“*[The practice change] made complete sense both logically and scientifically.*”—Ob-Gyn Physician
“*Oh, it was just accepted.”-* Ob-Gyn Physician
“*I mean, I'm always excited. Whenever there is kind of new evidence that's showing one, that we can have to either less frequently screen or maybe do it in a more patient-friendly manner, not having to use, for instance, as many brushes…and doing a pap smear. The switchover to one brush* vs. *two was…a nice kind of patient-friendly kind of approach, too.”—*Family Medicine Physician
Feasibility
“*…for the most part, everything went really well. There was no hiccups as far as I can recall. It was a pretty smooth transition.”—*Family Medicine Medical Assistant
“*…most of all, [the transition] was pretty smooth.”—*Ob-Gyn Physician
“*I feel the transition went very well. All things considered.”—*Family Medicine Physician
“*Frankly, it's less because we only have to pull one where [we] were pulling two. We were touching that cervix twice with that sticky, pointy brush. So, it's probably better.”—*Ob-Gyn Licensed Vocational Nurse
“*…as far as myself there was no hiccups. It seemed to go okay, and I didn't hear anything more than that. I think it was an easy transition. It wasn't something that was crazy difficult…we were already doing it. We just went from doing two specimens to down to one. We're, awesome. [laughing] No more having to write…number one, number two on the label, which one was the pap, which one was the HPV, and we don't do that anymore.”*—Family Medicine Medical Assistant
Ease of Practice Change
“*I think, comparably, this was the easiest transition. I feel it was explained very well. We knew what we were doing. We were ready for the change before it happened. So, I think out of all of the [practice] changes we've had, this was the easiest one.”—*Family Medicine Physician
“*I think it went really well…I think it was the best explained implementation of a new policy that I've had in 11 years of being here.”—*Family Medicine Physician
Themes: Initial Post-Implementation Barriers
** **Representative Quotes
Specimen Collection and Ordering
“*Everybody is at one brush now. But in the beginning, some doctors didn't want to change..we were guessing and making sure we had the right brushes.”***—**Ob-Gyn Licensed Vocational Nurse
“*The main learning curve was…for a few months…they didn't know which order to put in, abnormal or normal history. It was hard when they went in because they kept both orders in our system for some reason, and so the nurses, for the longest time, didn't know which order to put in, and I think they still kept both in there. And then…it was always do we do two or one..now our OB nurses obviously know, so it's been fine.”*—Ob-Gyn Physician
“*I don't know if it was just because of the way that the smart order set changed, and then included kind of separating out rather than just age based, but more age based and abnormal history. If that made more complicated for staff. But to be perfectly honest, looking at the smart set from a provider perspective, it doesn't seem to be too confusing.”*—Family Medicine Physician
Lack of Standardization in Training for Nurses and Physicians
“*…it always feels like doctors are told something else as opposed to [nurses]… and [when] we're trying to work together,…we're miscommunicating because we each got two different perspectives…on whatever we're supposed to be ordering…or the way we're supposed to put it in the system, stuff like that.”**—***Ob-Gyn Licensed Vocational Nurse
“*But then once we started to kind of implement, I did notice that I think we were not on the same page in regards to nurses, which are the ones who pend our orders and us.”-* Family Medicine Physician
“*Even for us, it still took some to try to understand it…the doctors had to go into detail. So, maybe that would've been better, so that us nurses understand the reasoning behind it, as well, before trying to educate somebody if we're not educated fully on it. In the future, when we do have changes, if they can train us or give us the information to be able to have as a reference, so when those questions get asked, or anticipate questions that our patients might ask…That would probably help, super helpful, so we're not stuck like, ‘Well, we don't know. Because they said so.’”-* Family Medicine Licensed Vocational Nurse
Themes: Persistent Post-Implementation Barriers
** **Representative Quotes
Delay Resulting Reflexive Cytology Labs
“*…sometimes I don't know what to do when I get a certain result back. And then the Pap…many times, when the HPV is positive, it takes forever for the actual pap to come back, because they're not running the pap until the HPV comes back…That's the only, I guess, patient dissatisfier…on their side, they're able to see HPV positive, and they're, “Why didn't you call me? What are we supposed to do?” And it takes sometimes a couple weeks before the pap comes back for you then to say, “Hey, here is your next step.*”—Ob-Gyn Physician
“*But what we do notice is there's still a little bit of an education gap for the patients when they're looking for their result…even though we tell them in the encounters and will often give them like patient instructions like this. Is HPV testing and this is what it means. And this is what to expect…[but] there's still a little bit of a holdover where people are going on to kp.org [the patient portal], and they don't understand how they're seeing the results…*”—Family Medicine Physician
“*The only issue in the beginning was when the HPV came back positive. The co-testing took a very long time to come back. I think it was taking four to six weeks, and a few times it didn't come back. So, it was just extra work on my part and a lot of extra anxiety on the patients' part because they saw a positive test, but they weren't given a diagnosis right away.*”—Internal Medicine Physician
Fear of Missed Cancer
“*…we're always having practice changes. Things are always changing. My concern was because I heard that the Pap wasn't getting run anymore, and me as someone that has a history of abnormal pap smears, that was concerning for me. I'm well what do you mean it's not being ran? And then when the doctor really explained it…I had a better understanding of it.*”—Family Medicine MA
“*I would say the concern probably from the older docs is, in the older days, there were other things that had caused cervical dysplasia, so from their aspect, it was, how can we not also check the cells? But for the most part I think everybody was accepting of that as the new way moving forward.*”—Ob-Gyn Physician
Patients Uninformed About the Practice Change
“*I feel it's almost we're not highlighting the change…we're leaving it to the providers to say if a patient is surprised, then it's the provider that then has to explain everything, which takes time and we often don't have time. We're not really prepping the patient for this. And I don't know if that's intentional.*”—Ob-Gyn Physician
“*But I think that a patient that's just coming in for routine screening, they feel you get the Pap, and they don't really recognize the difference that we're only running HPV where before we were running HPV and cytology.*”—Ob-Gyn Physician
“*I feel like we're not highlighting the change. Like we're leaving it to the providers to say, you know, like if a patient is surprised, then it's the provider that then has to explain everything, which takes time and we often don't have time. So, we're not really prepping the patient for this. And I don't know if that's intentional. Because sometimes, that's a big task as well, and it may cause more—like patients that, you know, they might just get upset seeing this. But so maybe that was the decision to just not address it until it comes up…I have never seen any patient education about it.*”—Ob-Gyn Physician
Theme: Fidelity to Interventions
** **Representative Quotes
“*I think based on the rollout, it happened, it went well. It was well organized. Information was shared appropriately. All the equipment was made available quickly, and we had immediate feedback from our staff or the lab.*”—Family Medicine Physician
“*I didn't get any feedback as far as challenges. When I was speaking to the coworker that was helping me in that time, he was just saying it was a lot more simpler for him, as well. And he appreciated the change.*”—Family Medicine Licensed Vocational Nurse
“*I think partially because of the discussion I was involved with prior to this rollout. It was information that I already had recorded in my mind, knowing that it was going to happen…When the new information came out, I didn't pay as much closer attention to it. Having said that, I think I recall our DA and our quality team, I believe it was our quality team or our Pap team leader, who had rolled out that information* via *email.*”—Ob-Gyn physician
“*I have not heard anything. In fact, I think most of us said, “Oh, good, I don't have to do it twice, but just one”…I think everybody loved it. So, you cut down the, you cut a few seconds of our life down. it's almost like a no-brainer. It's a better, easier process, so I have never heard of anything about it…*”—Family Medicine Physician
Theme: Use of Adaptations Across Sites
** **Representative Quotes
“*We stayed very true to form.*”—Ob-Gyn Medical Assistant
“*According to the instructions is how we do it.”—*Family Medicine Licensed Vocational Nurse
“*No, I think we did it the way it was intended to be done.”—*Family Medicine Physician
“*I think we rolled it out just how they asked. I don't think we made any changes.”—*Family Medicine Licensed Vocational Nurse
Theme: Recommended Refinement to the Practice Change Approach
** **Representative Quotes
Provider Training
“*I mean, we obviously trust that there is a group that is on the frontline of this, and really know the ins and outs. And so, we were just like okay, if this what they looked into and validated and then, you know, this is what we're going to do. Like, we have that trust in that. Because I know how everything, you know, within Kaiser, like they don't just make changes overnight. It's like a huge process. And so, for it to pass through that process, there have, you know, dozens of eyes have been on this. And so, it's not something that you—we question. But of course, the curious, the—you know? Intellectual curiosity is there, and people are like, huh, you know? And I feel like if they provided an opportunity maybe for a webinar that focused on the data and the science behind it? That that would have been very beneficial, and a lot of people would have been interested in that… A separate webinar on the medicine and the, you know, evidence behind it.”—*Ob-Gyn Physician
“*I think, you know, it's with any change. I think just making sure it's communicated and, like you know, like I said, everyone's a different learner. So, if it means, you know, visually kind of going through or doing this step by step and kind of walking through it rather than just like handing a paper, that makes a big difference”.*—Family Medicine Physician
“*I believe standardizing not only the nurses but also the doctors. Because I feel like everyone, again, works differently. But we…should stick to the same way of practicing…it's just the whole, let's be a team. Like everyone work the same…”—*Family Medicine Physician
Patient Education
“*I think that in terms of—and I don't know. I think the providers like—I don't know what is being shared with patients. Like maybe if they sent this to the patient, and actually, you know, obviously patient friendly format. But like, hey, you know? This is how we're doing this now, and 99.7% is caused by HPV, and explained it in patient language. I think that would help the providers. I don't know if that was done. I feel like—I mean, and I could be wrong. I don't know that the patients knew we were rolling this out.”—*Ob-Gyn Physician
“*I think more basic, simpler terms. And then pictures. Pictures always help, it seems, you know, where patients understand. Even multiple pictures for them to just better understand the concept of what they're trying to tell them.”—*Family Medicine Physician
“*But even the change, I wish we could have notified our patients first. Because they want to know what we're testing for.”—*Family Medicine Physician
“*I mean, now, thinking about it, it would've been nice if we had some information to hand out to our patients to better explain it, ourselves. Even for us, it still took some to try to understand it. Like, oh, okay. And the doctors had to go into detail. So, maybe that would've been better, so that us nurses understand the reasoning behind it, as well, before trying to educate somebody if we're not educated fully on it.”—*Family Medicine Licensed Vocational Nurse

**Table 3:** Participant recommendations to improve implementation of primary HPV cervical cancer screening.

Clinician training	Patient education
• Create a short (≈10 min) video for clinicians explaining the evidence for the practice change	• Inform patients of the practice change PRIOR to the rollout
• Plan for multiple reinforcement webinars post-rollout of the practice change	• Deliver materials to patients directly rather than relying on providers to offer them to patients during busy visits
• Communication and training opportunities targeting all staff should be standardized (e.g., nurses and physicians)	• Offer different modes of patient education (i.e., posters, handouts, clinician scripts)
• Provide communication about the practice change in (1) repeated and (2) multiple formats	• Educate patients about the sequencing of testing (e.g., a reflexive PAP is performed if the HPV test is positive) and the time required for processing tests (to avoid patient anxiety while they await their results)
• Account for retraining of new staff, float pool nurses and staff redeployments	• Remove patient instruction templates referring to PAP test from any patient-facing materials; use “*cervical cancer screening*' instead

#### Feasibility and acceptability of the practice change

Participants from both arms of the trial noted awareness, preparedness, and buy-in regarding the transition to primary HPV screening was high and that primary HPV screening was quicker and easier. Most clinicians understood the change was coming due to communication from the internal KPSC HPV Cervical Cancer Screening Task Force, and overall, the implementation reportedly “*…seemed to go okay*.” The general impression of the practice change was that it improved efficiency because teams would be collecting only one specimen as opposed to two *(“…only have to touch the cervix once”*) and was evidenced based (“*I don't remember anyone having any major concerns about this at all. Scientifically, I think we all kind of accepted that this made sense.*”). In hindsight, clinicians perceived it as an easier practice change than previous transitions at KP, given that most changes to practice add to the clinical workload, rather than reduced effort (*“And I don't know, I felt they did a better job than other times when changes have occurred.”*).

#### Fidelity to the practice change and implementation strategies

##### Fidelity to the primary HPV screening practice change.

Fidelity to the new HPV primary screening process was high and clinicians reported quickly shifting to the new process and using Pap reflexively according to guidelines (i.e., after a positive HPV result). Many participants reported that the new screening process was quicker and easier to conduct (*“It was pretty easy just to, “Okay, you're going to do one sample and one specimen container.” And that's it.”*). In addition, access to the clinician education (emails, webinars) and provision of correct supplies (i.e., exam tray set-up) were key facilitators for MAs and LVNs to conduct the screening:

“I know they gave a flyer because I saw the flyer on my desk. That was very self-explanatory..But that was enough…[to explain]…it's going to be the 2-in-1 in one vial and then showing us a little vial with a little scrape, a little brush…Instructions…”

##### Fidelity to the centrally-administered strategy.

Regarding the centrally-administered implementation, which included the core educational elements (e.g., emails to department chiefs to share with clinical teams, webinars, materials), participants reported general awareness and use of the implementation strategies, indicating a degree of fidelity to the centrally-administered strategy:

“So, I think the first, initial way that I found out about [the transition to primary HPV screening] was via an email…I think it even had a video to it and I didn't look at the video for a really long time, I don't think. I think I just let it sit in my inbox, but then I did look at it. And if you went through the video, it was super self-explanatory…I know that the actual ADAs [assistant department administrators] didn't really explain it to us and go over it until it was almost implemented.”

The dose of the implementation strategies was perceived as variable, with some participants remembering multiple strategies and others one or none *[“They were just told that here is the date we're switching to just doing one (specimen).”*] Overall, the strategies as delivered had an impact and allowed the clinical teams to make the transition smoothly (*“Actually, I think it went pretty smooth in our department. They did talk to us about it… we had a whole little meeting about it, maybe about two weeks prior to the big change.”*). However, the majority of participants were unsure of all of the details of the strategies (*“Yeah, emails, and I think it was mentioned in a meeting. Like, I don't remember the exact setting of…who talked about it and what they talked about, but I remember we had some education about it.”*), and some did not recall any at all. Some participants did note the timing of the implementation during COVID [*“I feel like (leadership) were more hands-on with the whole COVID and what's happening, and I felt like that's what they were focused on*.”].

##### Fidelity to the locally-tailored strategy

Within the local-tailored arm, where teams received additional resources that were tailored to their reported implementation needs, most participants could not recall receiving the locally tailored, patient- and clinician-facing materials: “*I don't recall them really touching the subject [HPV primary screening] again. But again, it was the pandemic, and there were other things they wanted to discuss.”* While few remembered receiving locally tailored material, participants agreed that getting follow-up and locally tailored materials would be a good reinforcement strategy for ongoing education.

#### Barriers to implementation

Initial barriers experienced immediately after the implementation were around specimen collection, which tests to order, and lab delays. One member of the nursing team explained:

“[In the beginning]…it was just hard for the nurses…they kept both orders in our system for some reason, and so the nurses for the longest time didn't know which order to put in.”

Another participant referenced the initial challenges some clinical teams experienced pending orders:

“Like I said, because me and my doctor, it took us a little while to finally figure out the correct way to order when it was a person who had an abnormal history vs. a person who had a normal history.”

There were several key barriers that persisted over time. The unanticipated increased length of time it took for laboratory to provide results for reflexive cytology tests (Pap) in cases where the primary HPV screening was positive increased patient anxiety and confusion. This in turn resulted in a reported uptick of patient email messaging and phone calls to clinicians and staff:

“Unfortunately, we're in this world where everything gets resulted and available to the patient instantly, which in this case is bad because to know you have a positive HPV and now have been told, ‘We need to wait for the Pap results,’ that's where the calls and confusion come in. So, we either stop giving them HPV results, but we can't, or you preempt by telling them, ‘If it's positive, we have to wait for the Pap. Then we'll let you know how we're going to triage you.’”

In addition, some participants feared missing cancer when transitioning from co-testing to primary HPV testing. This resolved for some members of the team after the practice change, but lingered for others, even months after the initial implementation period. One participant noted:

“I share an office with somebody. The two of us I remember were discussing like, ‘Oh, occasionally we'll have somebody who is HPV-negative but still have something on their cervical pathology.’ So, there was maybe a little concern that something would be missed… Honestly speaking, a lot of the LVNs don't know the answer to that…And I'll be honest, I still don't know the answer to that.”

On the other hand, patient pushback regarding the practice change was reportedly minimal. Clinicians largely attributed this to limited patient knowledge about the practice change as clinicians had switched their wording to “cervical cancer screening' rather than “Pap smear' when describing the screening service, and there had been little patient outreach about the practice change. As a result, many patients were unaware or unconcerned about the change unless their clinicians mentioned it (*“I do a lot of messages for my clinic providers and there are a lot of patients requesting their Pap results that I am unable to give and (were) not aware of the change until a provider mentioned it without explanation*.”). One MA remarked, “*It would help if every woman eligible for routine screening got an email or letter that explains the change automatically.”*

#### Use of adaptations across sites

The majority of participants did not report any specific adaptations to the practice change, rolling it out as the region requested *(“I think we did it exactly as prescribed..We didn't make any adaptations.”*)*.* One Ob-Gyn LVN explained how her team worked to approach the practice change cohesively and by design, *“…the team was pretty good about, obviously, if they weren't getting it right, then they would just come and ask questions, which was good.”*

#### Recommended refinement to the practice change approach

Participants offered recommendations for improving implementation focused on suggestions for patient education and clinician training. For instance, better education for patients about the sequencing of testing and the expected resulting timeframe was recommended to alleviate patient anxiety while waiting for reflexive cytology results. Other clinicians, such as the Ob-Gyn physician below, suggested it would be better to provide education materials to patients directly (e.g., through messages via the patient portal) rather than relying on clinicians to offer them to patients as clinicians have limited time and educational materials often get lost in message overload:

“I don't know what is being shared with patients…maybe if they sent this to the patient [in a] patient friendly format. This is how we're doing this now, and 99.7% is caused by HPV, and explained it in patient language. I think that would help the providers. I don't know if that was done.”

MAs and LVNs recommended standardized communication/training for nursing staff, perceiving they did not receive the same extent/quality of training as physicians (“*As screening nurses we learn about the process on collecting the specimen not why it is recommended. Only the doctors go to these seminars to learn the why's.”*). One LVN suggested that when it comes to training for a practice change like this, treat the nurses and physicians like a team:

“I would think you would just need a team. A team that's going to educate everybody. Not separate teams. Not a physician rolling it out and explaining it to the physicians and then another nurse rolling it out to the staff. Because obviously, they're not on the same page as to explaining or giving the information…say you had a team that was running…the HPV rollout and that same team explained it to the doctors and that same team explained it to the staff because then we know we're all getting the same message.”

## Discussion

Overall, participants reported smooth implementation and a high degree of fidelity to the new HPV primary screening process. The ease of implementation was often credited to the simple practice change of going from two clinical specimens to one. Initial barriers included getting comfortable with the new collection process and entering proper cervical cancer testing orders for patients with both routine and abnormal history. Ongoing challenges included some clinicians continued fear of missing cancers without co-testing, and patient lack of awareness regarding the practice change prior to implementation. An important but unanticipated barrier was the delay in results for the reflexing cytology (Pap) after a positive primary HPV test, resulting in patient anxiety about the positive HPV test while waiting for the reflexive cytology result. This may be a significant challenge for other health systems making the transition to primary HPV cervical cancer screening. This finding echoes those from a recent study from Australia, where national primary HPV screening was implemented starting in 2017. In the Australian study, key stakeholders in the implementation and use of primary HPV screening (clinicians, administrators, policy makers, pathology laboratories) were evenly divided on whether the implementation was successful (42). While overall support for program implementation was high, significant barriers were identified with a particular emphasis on feasibility. Barriers to feasibility included lack of knowledge regarding clinical guidelines and testing procedures at the clinician- and lab-level, errors in correspondence regarding results, and increased need for colposcopy. Although the overall uptake and acceptability of the primary HPV screening program was high in the KPSC setting in our study, the concerns regarding communication and understanding of reflexive cytology results was a significant issue.

Regarding fidelity to the implementation strategies, there was reported fidelity to the centralized educational strategy for clinicians (e.g., attending webinars, reading emails from department leadership, etc.) and these interventions appear to have been a strong facilitator of practice change. However, some participants reported low or no engagement with (or memory of) the educational resources provided via the centralized approach. In the local-tailored arm, few remembered the local-tailored resources provided, and it was difficult for participants to determine what was centralized and what was tailored at the local-tailored sites. Additionally, the few who could recall receiving specific materials generally didn't remember using them in their clinic. Interestingly, the data showed some contradiction regarding the local-tailored materials: participants agreed that receiving tailored follow-up materials and resources is a good approach that can help with process change (e.g., reinforcement); however, few remembered receiving or using them. The COVID pandemic likely affected recall, with several participants remarking that the pandemic was front and center such that educational efforts focused on other issues were not attended to or remembered. Additionally, the tailoring activities occurred after the centralized approach from the health system, potentially blurring the activities together in participant memory. It is also possible that the “dose' of the locally-tailored strategy (1–2 meetings with the local team, lack of in-person meetings for discussion and dissemination) was not enough to produce meaningful differences in fidelity or uptake/memory of tailored intervention materials.

Participant recommendations to improve implementation included educating patients prior to implementation about primary HPV screening and what patients should expect regarding the sequence of results in the event of a positive primary HPV test result. Within the context of other recent studies conducted in the U.S. and internationally, patient-facing education about primary HPV screening and interpreting HPV results should be a key element of implementation. In a recent qualitative study conducted in England, where a national primary HPV screening strategy was implemented, women reported confusion and multiple unanswered questions about both the HPV screening protocol and the meaning of the HPV result (43). This was particularly acute for women with lower educational attainment. Another study from England using a cross-sectional survey design found that women who received their first positive HPV result had high anxiety about cervical cancer (44). A 2015 survey study of U.S. women found that HPV primary screening was the least acceptable cervical cancer screening option (17). Increasing patients' knowledge about primary HPV testing for routine cervical cancer screening and interpretation of results—including the need for reflexive cytology for those with positive HPV tests—in advance of widespread implementation may help to reduce patient anxiety and decrease the burden on clinicians. Patient education about cervical cancer screening modalities has been shown to be effective; for example, a short video about preferred method and interval of screening was found to increase acceptance of screening modalities other than annual Pap smear in screening-eligible women (45).

We also found that nursing staff, including MAs and LVNs, strongly recommended standardized pre-implementation communication and training for all clinical staff; they perceived that they did not receive the same extent and quality of training as physicians/advanced practice providers. Training across clinical teams may be a key element of primary HPV screening implementation. This finding is echoed in a recent study of physicians, advanced practice providers, and MAs based in Indiana (U.S.) where cross-sectional survey findings illuminated limited acceptance and uptake of primary HPV screening, with only 3% of participants reporting using primary HPV testing and 50% willing to adopt it (15). In-depth interviews conducted as part of the study found high levels of uncertainty, knowledge gaps, and perceived limitations of primary HPV screening (45). Within the U.S., there is the added layer of complexity that U.S. guidelines currently recommend a suite of screening strategies with the new addition of primary HPV as an option [screening every 3 years with cytology alone, every 5 years with HPV testing alone or every 5 years with HPV testing in combination with cytology (co-testing) in women 30–65 years, based on risk level], European guidelines unequivocally recommend primary HPV screening (9). Recommending multiple options for screening may compound the difficulties of implementation in the U.S. context given the multiple options recommended in current clinical practice guidelines.

### Limitations

The primary limitations related to the interview results are potential selection bias and imperfect recall. Selection bias may have occurred because we were only able to interview a few members of any clinical team across sites and the perspectives of those interviewed may not be representative of the views of other team members who did not participate. Clinicians who completed interviews may be somewhat different compared to clinicians who did not respond. Imperfect recall likely occurred, as there was indication that some participants were unclear on what materials and resources they received from the centralized vs. the locally tailored strategy. In addition, all participants were from a single large integrated healthcare system (KPSC) which may limit the overall generalizability of results. However, other studies conducted both within the U.S. and internationally have identified similar themes, particularly around the need for coordinated education for both patients and clinicians.

In conclusion, despite challenges, the overall implementation of primary HPV screening had high acceptability, feasibility, and fidelity, and the centralized strategy appears to have been an effective implementation strategy for this practice change. Initial challenges to the rollout included specimen collection and primary HPV ordering issues within the electronic medical record. Other barriers included fear of missed cancer, variations in communication and training for nurses vs. physicians, and increased patient anxiety due to delays in results for reflexive cytology when the primary HPV test was positive. Our findings can be applied to other health systems and settings considering implementation, particularly those within the U.S. or with a similar health care model.

## Data Availability

The datasets presented in this article are not readily available because Individual-level data reported in this study involving human research participants are not publicly shared due to potentially identifying or sensitive patient information. Upon request, and subject to review, KPSC may provide the deidentified aggregate-level data that support the findings of this study. Anonymized data (deidentified data including participant data as applicable) that support the findings of this study may be made available from the investigative team in the following conditions: (1) agreement to collaborate with the study team on all publications, (2) provision of external funding for administrative and investigator time necessary for this collaboration, (3) demonstration that the external investigative team is qualified and has documented evidence of training for human subjects protections, and (4) agreement to abide by the terms outlined in data use agreements between institutions. Requests to access the datasets should be directed to Chun.R.Chao@kp.org.
