# Supplementary material for: Locally-tailored vs. centrally-administered strategies for implementation of primary human papillomavirus (HPV) screening in an integrated healthcare system: a qualitative research study
Source: Front Health Serv. 2025 Jul 15;5:1595934. doi: 10.3389/frhs.2025.1595934 (PMC12303904; doi:10.3389/frhs.2025.1595934)
Supplement: Supplementary file 2 [file Table1.docx]

**POST-IMPLEMENTATION EVALUATION GUIDE: QUALITATIVE**

**Introduction**: **Thank you so much for your willingness to talk with us today! We are interested in learning how to improve our approach to implementing new care processes within our region. In mid-2020, we changed our cervical cancer screening approach from co-testing for patients aged 30-65 with routine history to primary HPV testing. As a health system, we were coping with pressures from COVID during this change, as well as new cervical cancer risk-stratification guidelines issued in 2019 from the American Society for Colposcopy and Cervical Pathology (ASCCP). We would like you to think back to mid-2020 as the primary HPV testing was rolled out within your department, and consider what went well, what didn’t go well, and where we might make changes so that our next clinical workflow change goes smoothly.**

**REFLECTION**

1. Thinking back on the transition from co-testing to primary HPV testing, how was the overall experience?

**Prompts:**

- Do you have any recollection of receiving information from the HPV Cervical Cancer Screening Task Force or your clinical leadership (Chief/DA) regarding this transition?
- You may have attended one of the educational webinars about this change?
- How about training/orientation to a new menu of SmartSets for routine cervical cancer screening orders?

**FIDELITY**

1. When was the approximate date of rollout at your clinic?

**Prompts:**

- On July 17, 2020, as instructed by region? Another timeline?

***We are aware that there were changes to risk categories and recommended follow-up, but for the purposes of our discussion today, we are primarily interested in primary HPV screening for low-risk women aged 30-65 years.***

1. With that in mind, would you say your team fully adopted the change from co-testing to HPV primary testing for these low-risk women? Why or why not?
2. Since the change to primary HPV screening, have there been instances where you’ve still performed Pap tests at the initial screen? If so, what prompted the test?
3. Are you measuring primary HPV screening uptake in any way at your clinic?
4. What feedback have you received from your team members about the implementation of this practice change? From DAs? Nurses? Physicians?

**BARRIERS/FACILITATORS**

1. Overall, how easy or difficult was changing from co-testing to HPV primary testing?
2. What specific problems or challenges, if any, did you/your team encounter when implementing this practice change?

**Prompts:**

- How did your team overcome those challenges?
- Are there any challenges that have not been resolved to date? Please describe.

1. To what extent have you been provided with patient education resources or other resources for this practice change?

**Prompt:**

- How useful did you find these resources in general? Any barriers to use?
- What patient resources/additional patient resources would you have liked to receive, if any?

1. Overall, what has been your team’s approach to presenting this change to your patients?

**Prompt:**

- How are physician-nurse teams talking to patients about the change?

1. How have patients reacted to this practice change?

**Prompts:**

- What questions do patients typically have about the change?
- Do you document patient concerns in the chart? If so, always? Under what circumstances? Where is this documented?
- Do you have any patient stories you can share related to this change?

**COVID IMPACT**

1. Looking back, do you think clinicians may be less likely to change practices when under unusual stressors such as the COVID pandemic?
2. During the past year, in your experience were women more likely to get screened when they were already in the clinic (higher successful opportunity rate) because they did not want to have to come back and risk further COVID exposure?
3. Do you think women would be more likely to accept a screening method that requires less frequent screening because they don’t have to come into the medical centers and risk exposure to COVID?

**ADAPTATIONS**

1. Did you/your team make any changes or adaptations to the recommended care processes required to change from co-testing to HPV primary testing? If so, please explain the adaptation.

- **Ex. adaptations** – still co-testing; extended timeline, how talking or presenting to patients about the change, etc.

1. Who championed this practice change in your clinic? What steps did they take to promote the change?

**SUSTAINABILITY/SCALABILITY**

1. Thinking about the HPV primary testing materials (patient handouts, webinar education, etc.), does using these in your regular clinical practice feel familiar/routine?
   1. *Probes*: Do you think you will continue to use these materials/approaches? Perhaps just one type?
   2. Was it easy to integrate into your workflow; why or why not?
   3. Overall do you think you would continue using this approach with your patients?
2. How well do you think this approach would work in other KP regions [**INTERVIEWER**: Include detail on tailored vs centralized based on site]
3. Could you see other health care systems or clinics using this approach? Why or why not?

**SUGGESTIONS**

1. Overall, to what extent do you feel you and your clinical team received enough support and/or education for this practice change?

**Prompts:**

- Were sufficient resources provided from the beginning to make a smooth transition?

1. What suggestions, in addition to any you have already mentioned, would you recommend improving the rollout of a similar practice change in KPSC? Specific suggestions of what could have streamlined the process would be very helpful.

***End of Interview: Thank you very much for your responses to our questions and participation in this interview. Do you have anything else you would like to add to the discussion before we conclude the interview?***
